# Data showing the circumvention of oxaliplatin resistance by vatalanib in colon cancer

**DOI:** 10.1016/j.dib.2016.02.064

**Published:** 2016-03-02

**Authors:** Kenneth K.W. To, Daniel C. Poon, Yuming Wei, Fang Wang, Ge Lin, Li-wu Fu

**Affiliations:** a School of Pharmacy, Faculty of Medicine, The Chinese University of Hong Kong, Hong Kong, China; b State Key Laboratory of Oncology in South China, Cancer Center, Sun Yat-Sen University, Guangzhou, China; c School of Biomedical Sciences, Faculty of Medicine, The Chinese University of Hong Kong, Hong Kong, China

**Keywords:** Vatalanib, Tyrosine kinase inhibitor, Oxaliplatin, Platinum drugs, Chemoresistance

## Abstract

We have recently reported that vatalanib, an orally active small molecule multi-tyrosine kinase inhibitor (Hess-Stumpp et al., 2005 [1]), can sensitize multidrug resistant (MDR) colon cancer cells to chemotherapy under hypoxia by inhibiting two MDR transporters ABCB1 and ABCG2 (To et al., 2015 [2]). This data article describes the possible circumvention of resistance to specifically platinum (Pt)-based anticancer drugs by vatalanib via inhibition of two other efflux transporters ABCC2 and ATP7A. Data from the flow cytometric transporter efflux assay showed specific inhibition of ABCC2 activity by vatalanib in stable transfected cells and ABCC2-overexpressing oxaliplatin-resistant colon cancer cells HCT116/Oxa. We also performed the transporter ABCC2 ATPase assay and showed an increase in ATP hydrolysis by ABCC2 in the presence of vatalanib. ATP7A mRNA expression was also shown to be upregulated in HCT116/Oxa cells. Vatalanib was shown to suppress this upregulated ATP7A expression. Data from the cellular Pt accumulation assay showed a lower Pt accumulation in HCT116/Oxa cells than the parental sensitive HCT116 cells. Vatalanib was shown to increase cellular Pt accumulation in a concentration-dependent manner. Combination of oxaliplatin and vatalanib was shown to restore the suppressed apoptosis in HCT116/Oxa cells.

**Specifications Table**

**Table:** 

Subject area	*Biology*
More specific subject area	*Chemoresistance, Platinum-based anticancer drugs, Tyrosine kinase inhibitor*
Type of data	*Table, text file, graph, figure*
How data was acquired	*Flow cytometry (LSRFortessa Cell Analyzer (BD Biosciences)), Real-time PCR (LightCycler 480 (Roche)), Inductive coupled plasma-optical emission spectrometer (Optima 4300DV (Perkin Elmer))*
Data format	*Analyzed*
Experimental factors	*HCT116 and its oxaliplatin-resistant subline HCT116/Oxa were treated with oxaliplatin or its combination with vatalanib.*
Experimental features	*Vatalanib-treated cells were subjected to ABCC2 efflux assay or cellular platinum accumulation assays. HCT116 and its oxaliplatin-resistant subline HCT116/Oxa subline treated with oxaliplatin in the presence or absence of vatalanib were processed for real time PCR analysis and annexin V apoptosis assay.*
Data source location	*School of Pharmacy, The Chinese University of Hong Kong, Hong Kong SAR, China*
Data accessibility	*Data are provided in this article*

## Value of the data

• Data show the circumvention of platinum resistance by combination of oxaliplatin and vatalanib in an oxaliplatin-resistant human colon cancer cell line HCT116/Oxa.
• Data show the upregulation of two efflux transporters (multidrug resistance-associated protein 2 (ABCC2) and copper efflux transporter (ATP7A)) in oxaliplatin-resistant colon cancer cells (HCT116/Oxa).
• Data show the inhibition of ABCC2 transporter activity by vatalanib.
• Data demonstrate the downregulation of ATP7A expression by vatalanib.
• The data may be valuable for future studies investigating the beneficial cytotoxic effect by combining vatalanib and platinum-based anticancer drugs in chemotherapy.

## 1 Data

We have recently reported that vatalanib, an orally active small molecule multi-tyrosine kinase inhibitor [1], can sensitize multidrug resistant (MDR) colon cancer cells to chemotherapy under hypoxia by inhibiting two MDR transporters ABCB1 and ABCG2 [2].

**Fig. 1:**
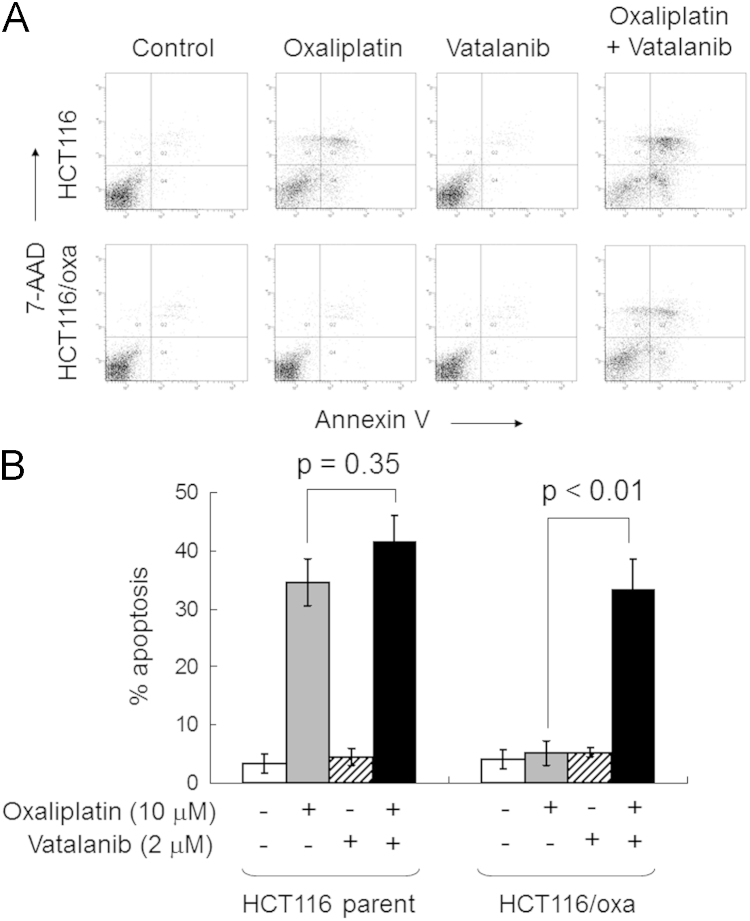
Vatalanib sensitized oxaliplatin-resistant HCT116 to oxaliplatin-induced apoptosis. (A) HCT116 parental cell line and its oxaliplatin-resistant subline were exposed to oxaliplatin (10 μM), vatalanib (2 μM) alone, or their combination for 48 h before harvest for apoptosis assay. A representative set of data from three independent experiments is shown. (B) Summary of apoptosis assay data from three independent experiments. Data are presented in histogram as means±SD.

**Fig. 2:**
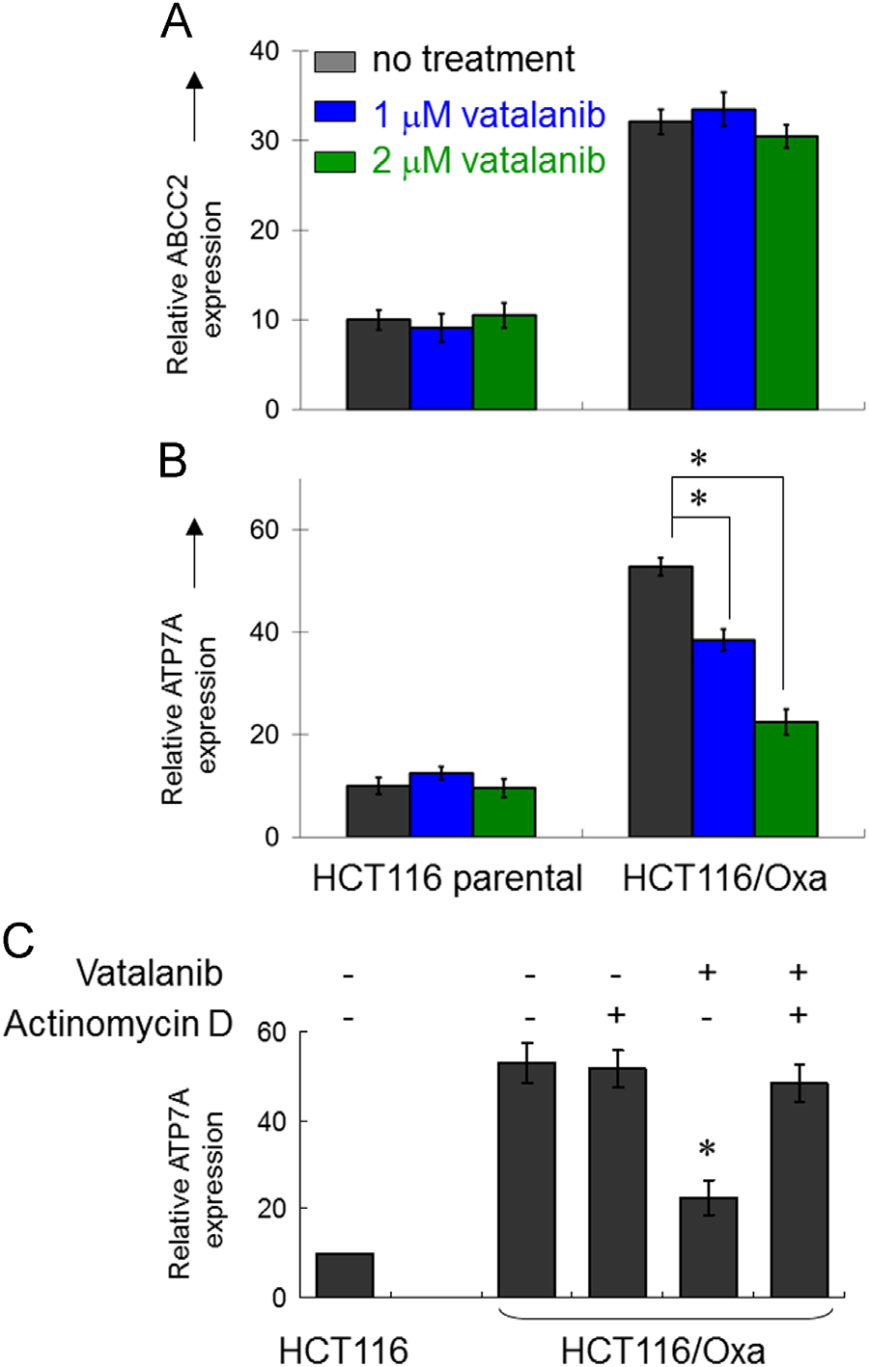
mRNA expression of ABCC2 (A) and ATP7A (B) in parental HCT116 and oxaliplatin-resistant HCT116/Oxa cells treated with or without vatalanib (1 or 2 μM, 24 h). Relative mRNA expression of the transporters are shown after normalization with β-actin. The expression in the parental HCT116 cells was set as 10 for comparison. * *p*<0.05, compared with untreated cells. (C) Real-time RT-PCR analysis of the downregulation of ATP7B in HCT116/Oxa cells by vatalanib with or without a 4-h pretreatment of actinomycin D (5 μg/mL).

**Fig. 3:**
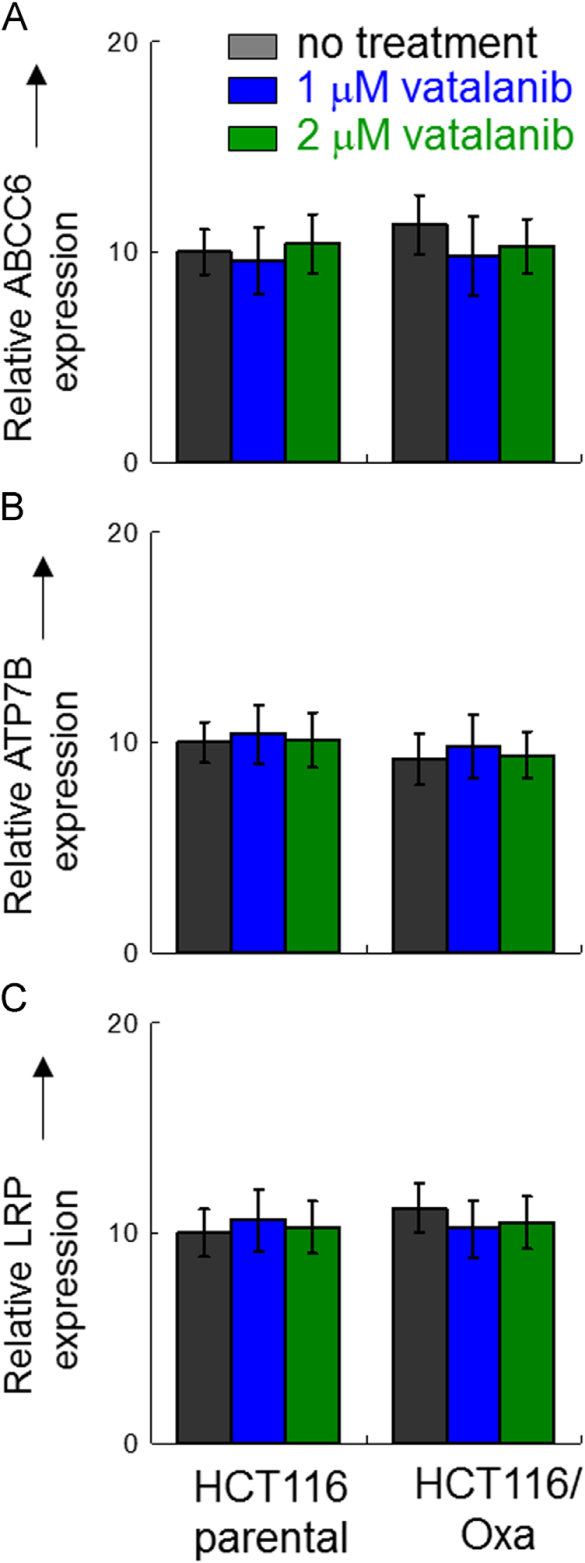
mRNA expression of other transporters implicated in Pt resistance: ABCC6 (A), ATP7B (B) and LRP (C) in parental HCT116 and oxaliplatin-resistant HCT116/Oxa cells treated with or without vatalanib (1 or 2 μM, 24 h). Relative mRNA expression of these efflux transporters are shown after normalization with β-actin. The expression in the parental HCT116 cells was set as 10 for comparison. No statistical significant difference was observed between the treatment groups.

**Fig. 4:**
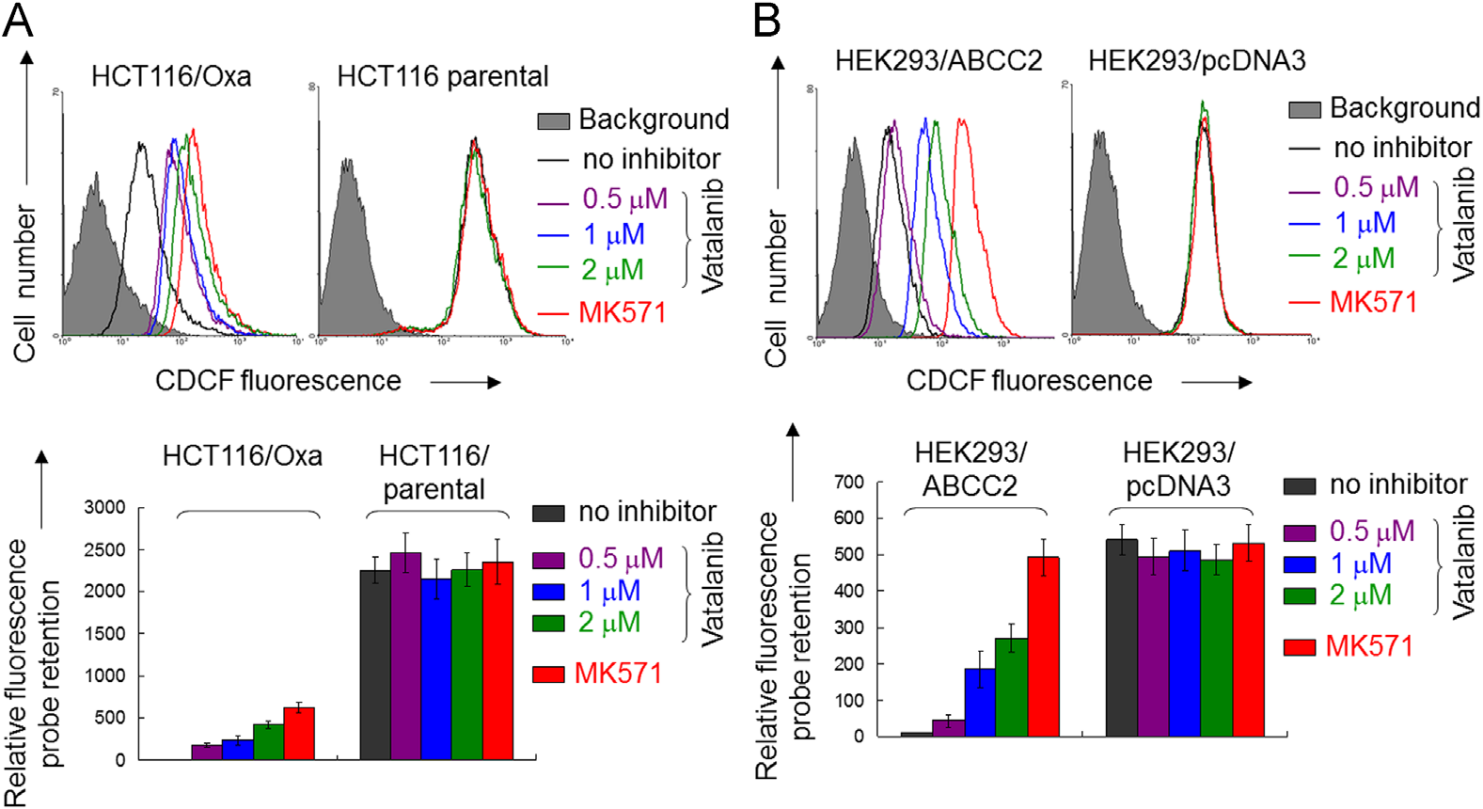
Inhibition of ABCC2-mediated efflux of fluorescent probe substrate CDCF by vatalanib in ABCC2-overexpressing HCT116/Oxa cells (A) and ABCC2 stably-transfected HEK293/ABCC2 cells (B). Data obtained in the HCT116 parental cells and the backbone vector transfected HEK293/pcDNA3 cells is also shown as control for comparison. Top panel: Representative efflux histogram from three independent experiments are shown. Bottom panel: The relative fluorescent probe retention was also quantified by setting the value in transporter-overexpressing cells in the absence of vatalanib as 10.

**Fig. 5:**
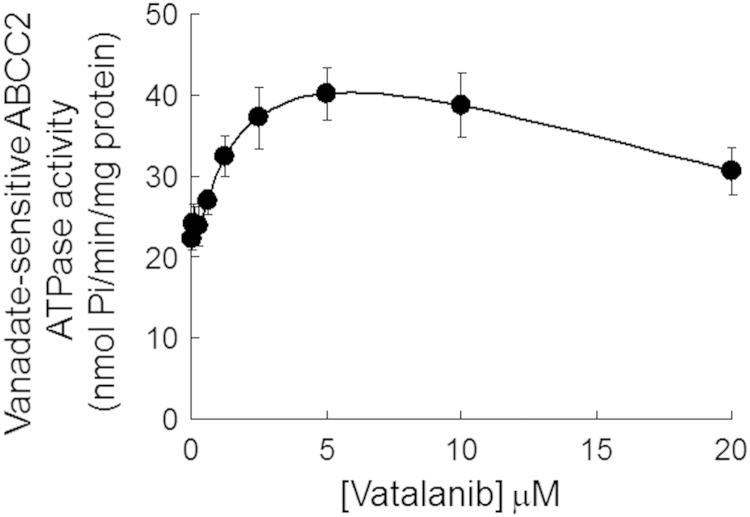
Effect of vatalanib on the ATPase activity of ABCC2. The vanadate-sensitive ATPase activity of ABCC2 in the recombinant transporter proteins obtained from cell membrane fraction was determined at different concentrations of vatalanib. ATP hydrolysis was monitored by measuring the amount of inorganic phosphate released using a colorimetric assay. In the presence of vatalanib, all of the stimulated ABCC2 ATPase activity was significantly different from the basal level (*p*<0.05).

**Fig. 6:**
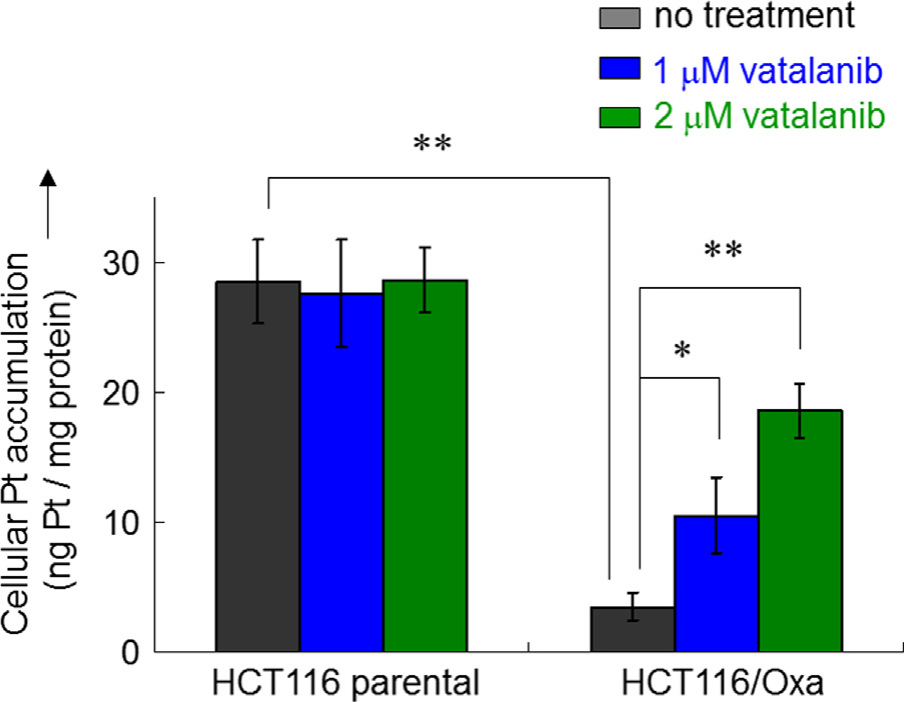
Cellular Pt accumulation in oxaliplatin (100 μM, 4 h)-incubated HCT116 and HCT116/Oxa cells in the presence of 1 or 2 μM vatalanib. In pilot experiments, drug accumulation was found to be increased in a linear manner with increasing drug concentration up to at least 400 μM. Cell lysates obtained were subjected to concentrated HNO_3_ digestion before analysis by Inductively Coupled Plasma Optical Emission Spectroscopy (ICP-OES). The absorbance of Pt at 265.95 nm was used for quantification. Protein concentration of the cell lysate was measured separately by the Bradford method for normalization purpose.

The data available in this paper are: (a) apparent circumvention of oxaliplatin resistance in HCT116/Oxa cells by combination of vatalanib and oxaliplatin (Table 1 and Fig. 1); (b) reduction of the upregulated ATP7A expression in resistant HCT116/Oxa cells by vatalanib (Fig. 2, Fig. 3); (c) inhibition of ABCC2-mediated drug efflux in resistant HCT116/Oxa and ABCC2 stably-transfected HEK293/ABCC2 cells by vatalanib (Fig. 4); (d) increase in ABCC2 ATPase activity by vatalanib (Fig. 5); and (e) increase in cellular Pt accumulation in resistant HCT116/oxa cells by vatalanib (Fig. 6).

## 2 Experimental design, materials and methods

### 2.1 Materials

5(6)-Carboxy-2′,7′-dichlorofluorescein diacetate (CDCFDA) was purchased from Biotium Inc. (Hayward, CA, USA). MK571 was obtained from Tocris Bioscience (Bristol, UK). Oxaliplatin was purchased from Acros Organics (Thermo Fisher Scientific, New Jersey, USA). Vatalanib was purchased from Selleckchem (Houston, TX, USA).

### 2.2 Cell culture

The human embryonic kidney cell line HEK293 and its stable pcDNA3- and ABCC2-transfected sublines were used to demonstrate the specific effect of vatalanib on ABCC2. The pcDNA3-transfected HEK293 cells were kind gift obtained from Dr. Susan Bates (National Cancer Institute, NIH, Bethesda, MD, USA). ABCC2 plasmid DNA for stable transfection was purchased from GeneCopeia (Rockville, MD, USA). The transfected cells were cultured in complete DMEM medium supplemented with 2 mg/mL G418. Oxaliplatin resistance was induced in the human colon cancer cell lines HCT116 (maintained in complete RPMI1640 medium) by prolonged incubation in progressively increasing concentration of oxaliplatin. Individual clones were obtained during the process and they were evaluated for sensitivity to oxaliplatin. The most resistant clone was used in the study (IC_50_=69.6±7.3 μM) (IC_50_ of oxaliplatin in parental HCT116 cells was 1.45±0.26 μM). Characterization of the HCT116/Oxa cells revealed an increase in expression of the efflux transporters (ABCC2 and ATP7A) (Fig. 2), a reduction in drug accumulation (Fig. 5), and elevated intracellular GSH content (Section 2), relative to the parental HCT116 cells.

### 2.3 Growth inhibition assay

The growth inhibitory effect of oxaliplatin and vatalanib was evaluated by the sulforhodamine B assay as described previously [3]. For the combination drug treatment, drugs were given simultaneously for 72 h.

### 2.4 Flow cytometric analysis of ABCC2 transporter activity

A flow cytometry-based assay was employed to study the inhibition of ABCC2 transport activity by vatalanib in an ABCC2 stable transfected HEK293 cells and an ABCC2-overexpressing oxaliplatin-resistant HCT116 colon cancer cells as described previously with minor modification [2]. The non-fluorescent membrane-permeable probe 5(6)-carboxy-2′,7′-dichlorofluorescein diacetate (CDCFDA) was used. After getting into the cells, CDCFDA is hydrolyzed to give the fluorescent 5(6)-carboxy-2′,7′-dichlorofluorescein (CDCF), which has been shown to be a specific substrate for ABCC2 [4]. The retention of the fluorescent CDCF in the cells is therefore used to indicate the efflux activity of ABCC2 in the cells. The cells were incubated with 0.5 μM CDCFDA and vatalanib at various concentrations at 37 °C and 5% CO_2_ for 30 min. Subsequently, the cells were washed with cold complete medium and then incubated for 1 h at 37 °C in CDCFDA-free medium continuing with the same concentration of vatalanib to obtain the efflux histogram. Inhibitors of the transporter ABCC2 is expected to shift the inhibitor/efflux histogram to the right, indicating retention of the fluorescent substrate in the cells. Inhibitors specific for ABCC2 (MK571 (25 μM)) were used as control for comparison. Samples were analyzed on an LSRFortessa Cell Analyzer (BD Biosciences, San Jose, CA, USA). CDCF fluorescence was detected with a 488-nm argon laser and a 530-nm bandpass filter. At least 10,000 events were collected for all flow cytometry studies. Cell debris was eliminated by gating on forward versus side scatter and dead cells were excluded by propodium iodide staining. All assays were performed in three independent experiments.

### 2.5 ABCC2 transporter ATPase assay

The effect of vatalanib on the vanadate-sensitive ATPase activity of ABCC2 in cell membrane prepared from High-Five insect cells was measured by using the BD Gentest ATPase assay kit (BD Biosciences) according to the manufacturer’s instructions.

### 2.6 Cellular Pt accumulation

Briefly, ABCC2-stable transfected HEK293 cells and ABCC2-overexpressing human colon cancer HCT116 cells were incubated with 100 μM oxaliplatin at 37 °C for 4 h. In pilot experiments, drug accumulation was found to be increased in a linear manner with increasing drug concentration up to at least 400 μM. Afterward, the cells were washed twice with ice-cold PBS and then harvested in NETN buffer (100 mM NaCl, 20 mM Tris HCl (pH 8.0), 0.5 mM EDTA, 0.5% NP-40), sonicated, and subjected to concentrated HNO_3_ digestion before analysis by Inductively Coupled Plasma Optical Emission Spectroscopy (ICP-OES) (Perkin Elmer Optima 4300DV, UK). The absorbance of Pt at 265.95 nm was used for quantification. Protein concentration of the cell lysate was measured separately by the Bradford method for normalization purpose.

### 2.7 Reverse transcription and quantitative real-time PCR

Real-time quantitative reverse transcription PCR was performed as described previously [2] to evaluate the expression of ABCC2, ABCC6, ATP7A, ATP7B and LRP in HCT116 and HCT116/Oxa cells treated with or without vatalanib.

### 2.8 Annexin V apoptosis assay

HCT116 parental cell line and its oxaliplatin-resistant subline HCT116/Oxa were grown on a 60-mm tissue culture dish at a density of about 2.0×10^5^ cells/well. They were treated for 48 h with 10 μM oxaliplatin in the presence or absence of 2 μM vatalanib. At the end of the treatment, both floating and attached cells were collected and washed twice with ice-cold phosphate buffer solution. The extent of apoptosis was determined by using the APC annexin V apoptosis kit (BD Bioscience, San Jose, CA, USA) according to the manufacturer’s instructions. Cells positive for both annexin V and 7-AAD were considered apoptotic.

### 2.9 Determination of cellular GSH content

Cellular GSH content was determined in parental HCT116 and oxaliplatin-resistant HCT116/Oxa cells by the GSH-Glo Glutathione assay kit (Promega, Madison, WI, USA). Protein concentration of the cell lysate was measured separately by the Bradford method for normalization purpose.

### 2.10 Data analysis and statistics

All experiments were repeated at least three times. The statistical software SPSS16.0 (IBM, Armonk, NY, USA) was used for data analysis. Statistical significance was determined at *p*<0.05 by the Student’s *t*-test.

## Figures and Tables

**Table 1:** Anticancer activity (IC_50_, μM) of oxaliplatin and its combination with vatalanib in a pair of parental and oxaliplatin-resistant human colon cancer cell lines (HCT116 and HCT116/Oxa, respectively), and ABCC2 stably-transfected HEK293/ABCC2 and its backbone vector transfected HEK293/pcDNA3 cell lines.

Cell line	IC_50_ of oxaliplatin±SD (fold resistance)		
	Oxaliplatin	Oxaliplatin+vatalanib (1 μM)	Oxaliplatin+vatalanib (2 μM)
HCT116 parental	1.45±0.26 (1)	1.61±0.21 (1.1)	1.32±0.23 (0.9)
HCT116/Oxa	69.6±7.3 (48)	23.5±6.9 (23)⁎	8.51±0.65 (5.9)⁎
HEK293/pcDNA3	0.51±0.35 (1)	0.63±0.29 (1.2)	0.59±0.17 (1.2)
HEK293/ABCC2	12.6±1.5 (25)	8.52±1.15 (17)⁎	3.55±0.86 (7.0)⁎

⁎ *p*<0.05, difference from oxalipatin alone in the resistant cells.
